# High Doses, High Standards: An Audit of Physical Health Monitoring in Patients Who Are Prescribed High Dose Antipsychotic Therapy Following Migration to an Electronic Health Record System

**DOI:** 10.1192/bjo.2026.11681

**Published:** 2026-06-30

**Authors:** Catherine Kerr, Helen Ford

**Affiliations:** 1 Northern Ireland Medical and Dental Training Agency, Belfast, United Kingdom; 2 Northern Health and Social Care Trust, Antrim, United Kingdom

## Abstract

**Aims:**

High dose antipsychotic therapy (HDAT) is associated with an increased incidence of physical health complications, typically cardiac and metabolic, due to the dose dependent side effect profile of antipsychotic medication.

Since the migration to a fully electronic health record (EHR), Epic, in November 2024 there has not been a streamlined or consistent approach to the completion of physical health monitoring within this patient cohort in our Community Mental Health Team.

This project aimed to evaluate and improve adherence to HDAT monitoring guidelines to reduce the risk of HDAT-related physical health complications.

**Methods:**

We screened our caseload of 896 patients and conducted a baseline audit in January 2026 against National Institute for Clinical Excellence (NICE) Clinical KnowledgeSummary (CKS) standards for antipsychotic monitoring (revised January 2026), with a target of 100% compliance.

We also measured the use of two functions of Epic, the ‘FYI Flag’ which is visible on the opening screen of each patient’s electronic chart and the ‘Therapy Plan’ which creates orders for appointments and specified investigations at defined intervals.

We then implemented change by ensuring all patients had an ‘FYI Flag’ for HDAT and an active HDAT ‘Therapy Plan’.

**Results:**

19 patients (2% of the caseload) were currently prescribed HDAT.

At baseline (January 2026), compliance with monitoring was 26%. 21% of patients had an FYI flag on their chart and 26% had HDAT therapy plans in place.

On reaudit (February 2026), compliance with monitoring was 58%. 100% of patients had an FYI flag on their chart and 100% had HDAT therapy plans in place.

Of the 42% of patients who had incomplete or outdated monitoring, 100% had an appointment scheduled for investigations to be completed. The majority of appointments were generated from the recently implemented therapy plans.

The most common reason for incomplete monitoring was investigations falling outside the recommended timeframe. The most commonly omitted components of an otherwise up-to-date set were prolactin and ECG completion.

**Conclusion:**

Adherence to HDAT monitoring guidelines was initially poor but improved substantially over a short period following targeted digital interventions.

This project demonstrates how the use of EHR functionality can efficiently improve patient care. We are optimistic that the rate of monitoring will continue to rise through the implementation of ‘Therapy Plans’ which electronically generate prompts for investigation completion and appointment booking.

We plan to continue this project with ongoing Plan–Do–Study–Act cycles to reach our target of 100% and demonstrate sustained improvement.

